# Laboratory findings and a combined multifactorial approach to predict death in critically ill patients with COVID-19: a retrospective study

**DOI:** 10.1017/S0950268820001442

**Published:** 2020-06-30

**Authors:** Q. Liu, N. C. Song, Z. K. Zheng, J. S. Li, S. K. Li

**Affiliations:** Department of Thoracic Surgery, Union Hospital, Tongji Medical College, Huazhong University of Science and Technology, Wuhan, 430022, China

**Keywords:** Blood urea nitrogen (BUN), coronavirus disease 2019 (COVID-19), D-dimer (DD), death, lymphocyte ratio

## Abstract

To describe the laboratory findings of cases of death with coronavirus disease 2019 (COVID-19) and to establish a scoring system for predicting death, we conducted this single-centre, retrospective, observational study including 336 adult patients (≥18 years old) with severe or critically ill COVID-19 admitted in two wards of Union Hospital, Tongji Medical College, Huazhong University of Science and Technology in Wuhan, who had definite outcomes (death or discharge) between 1 February 2020 and 13 March 2020. Single variable and multivariable logistic regression analyses were performed to identify mortality-related factors. We combined multiple factors to predict mortality, which was validated by receiver operating characteristic curves. As a result, in a total of 336 patients, 34 (10.1%) patients died during hospitalisation. Through multivariable logistic regression, we found that decreased lymphocyte ratio (Lymr, %) (odds ratio, OR 0.574, *P* < 0.001), elevated blood urea nitrogen (BUN) (OR 1.513, *P* = 0.009), and raised D-dimer (DD) (OR 1.334, *P* = 0.002) at admission were closely related to death. The combined prediction model was developed by these factors with a sensitivity of 100.0% and specificity of 97.2%. In conclusion, decreased Lymr, elevated BUN, and raised DD were found to be in association with death outcomes in critically ill patients with COVID-19. A scoring system was developed to predict the clinical outcome of these patients.

## Introduction

Since the outbreak of disease caused by a new virus in Wuhan, China in December 2019, hundreds of thousands of people have been infected with the virus. As of March 2020, the cumulative number of confirmed diagnoses on the Chinese mainland has been over 80 000, but there has been no new domestically transmitted case reported for several days, which indicates that the epidemic in China has been basically controlled. However, the viral disease has swept into at least 190 countries and killed tens of thousands of people. Over the last few weeks, the number of cases of this disease outside China has increased dozens of times. Therefore, the World Health Organization (WHO) had made an assessment on 11 March that the disease could be characterised as a pandemic [1].

The pathogen of the disease was first isolated by Chinese scientists on 7 January 2020, and was identified as a new type of coronavirus [2], which was initially known as 2019-nCoV and recently named SARS-CoV-2 [3]. It has the characteristics of a typical coronavirus family and belongs to the beta coronavirus 2b lineage. Although the initial epidemiological investigation suggested that it was transmitted to humans through wildlife, the distinct human-to-human transmission phenomenon has been confirmed in subsequent studies [4]. Full-genome sequencing and phylogenic analysis suggest that it is similar in structure to severe acute respiratory syndrome (SARS) virus, and mainly causes respiratory tract infections in humans [5], which was designated as coronavirus disease 2019 (COVID-19) later by WHO.

Several descriptive studies have confirmed that most infected patients were mildly symptomatic or even asymptomatic [6, 7]. However, there were still many patients who developed severe pneumonia or even death [8, 9]. The relevant indicators of this part of the patients, especially the patients who died, need to be of significant concern. A data-driven analysis stated the Hubei province (except Wuhan) has an estimated case-fatality rate (CFR) of 1.41% [10], which was also confirmed by a recent retrospective study involving 1099 patients [11]. However for critically ill patients, the mortality rate is much higher. A study from Jin Yin-tan Hospital of Wuhan compared the critically ill and non-severe patients, and the results showed that the mortality of critically ill patients with COVID-19 was considerable [9]. However, there is still no specific and effective treatment except for meticulous supportive care for this disease. No solid evidence proved any antiviral agents could improve outcomes in COVID-19. Even lopinavir−ritonavir treatment, previously thought to be effective, has been shown by studies that no benefit was observed in hospitalised adult patients with severe COVID-19 [12]. In such cases, critically ill patients could rapidly develop acute respiratory distress syndrome (ARDS), organ dysfunction and other serious complications and eventually death. Therefore, it is significant to estimate risk factors for severe disease and death, so that we could more efficiently focus on medical resources to treat patients who may have poor prognoses in efforts to reduce mortality.

Although there have been previous articles describing the clinical course of the disease or some factors that predicted death [13–15] because many patients were still in the middle stage of the disease at the time of the study and did not reach their clinical outcome, the collected clinical data were inevitably incomplete, and the results were less accurate. Under the situation that the epidemic is basically under control, most patients have their definite outcomes, either cured or died, so that the research would be more accurate, which was why we conducted this research study at this time. In addition, we used a combined predictive system with several variables, which also ensures the validity of the prediction.

In this research study, we collected the clinical data of patients with COVID-19 admitted in the hospital, encompassing laboratory indexes and their clinical outcomes (cure or death). We aimed to describe laboratory findings of cases of death, compare them with cured patients, and finally design a multifactor prediction model, which is expected to provide early identification for patients with clinically severe COVID-19.

## Methods

### Patients

**Fig. 1.**
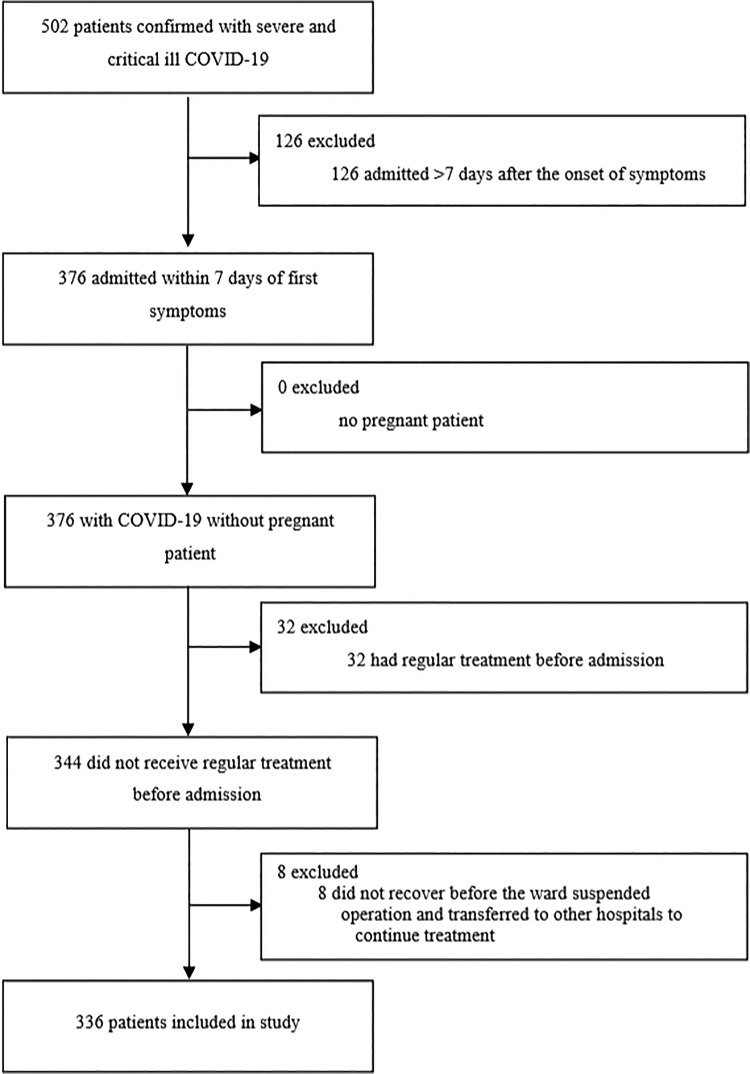
Study flow diagram. COVID-19, 2019 novel coronavirus disease.

This single-centre, retrospective, observational study included adult patients (≥18 years old) with severe or critically ill COVID-19 admitted in two wards of Union Hospital, Tongji Medical College, Huazhong University of Science and Technology in Wuhan. All patients were diagnosed with COVID-19 according to WHO interim guidance and they all had a definite outcome (death or discharge) between 1 February 2020 and 13 March 2020 (Fig. 1).

### Data collection

Demographical data, laboratory indexes, medical history, underlying diseases and outcome data were extracted from the blood screening test and electronic medical records. All data were examined by one independent physician through the patient's paper charts. The study was conducted in accordance with the principles of the Declaration of Helsinki. Informed consent was not obtained from patients as all data were retrieved retrospectively from the laboratory testing information system and no additional blood samples were taken. This study was approved by the ethics review board of Wuhan Union Hospital.

### Definition

The presence of SARS-CoV-2 in respiratory specimens was detected by next-generation sequencing or real-time RT-PCR methods to confirm the diagnosis of COVID-19 as described in the previous article [16]. Underlying diseases included hypertension, cardiovascular disease, diabetes, malignancy, cerebrovascular disease, chronic obstructive pulmonary disease (COPD), chronic kidney disease, chronic liver disease, organ transplant history and undergoing general anaesthesia surgery within 1 month. The duration from the onset of disease to hospital admission was recorded. The neutrophil ratio (Neur), is the percentage of neutrophils to the total number of all white blood cells (WBCs). Similarly, the lymphocyte ratio (Lymr) is the number of lymphocytes as a percentage of the number of WBCs.

### Statistical analysis

Statistical analyses were performed by SPSS 17.0 (SPSS Inc, Chicago IL, USA). Continuous and categorical variables were presented as median, interquartile range (IQR) and *n* (%), respectively. The Mann−Whitney *U* test, *χ*^2^ test or Fisher's exact test were used. Binary logistic regression was conducted further. We used both single variable and multivariable logistic regression to verify those factors. Variables with statistically significant differences in the single variable analysis were included in the multivariable analysis, and several variables with potential for bias were excluded. This was followed by a backwards stepwise logistic regression. The final model was used to predict death and that sensitivity/specificity/ROC curves were produced. A *P*-value < 0.05 was considered statistically significant.

**Table 1.** Characteristics of all patients and results of single variable logistic regression analysis

	Total (*n* = 336)	Survivor (*n* = 302)	Non-survivor (*n* = 34)	OR (95% CI)	*P*
Sex (M/F)	169/167	148/154	21/13	1.681 (0.812–3.479)	0.162
Age (year)	65 (51–69)	64 (51–68)	74 (64–78)	1.090 (1.048–1.134)	<0.001
Comorbidity (with/without)	182/154	165/137	17/17	1.204 (0.592–2.448)	0.607
Smoker	18 (5.4%)	15 (5.0%)	3 (8.8%)	1.839 (0.956–1.782)	0.329
Drinker	20 (6.0%)	18 (6.0%)	2 (5.9%)	4.656 (0.820–26.425)	0.082
Days	2 (1–4)	2 (1–4)	3 (1–5)	1.123 (0.928–1.357)	0.232
WBC (g/l)	5.93 (4.71–7.41)	5.74 (4.52–7.01)	10.27 (7.30–18.10)	1.857 (1.477, 2.336)	<0.001
Lym (g/l)	1.30 (0.89–1.71)	1.36 (1.01–1.72)	0.42 (0.25–0.60)	0.012 (0.003, 0.047)	<0.001
Neu (g/l)	3.87 (2.77–5.01)	3.48 (2.73–4.75)	9.39 (6.20–17.11)	1.307 (1.189–1.437)	<0.001
Lymr (%)	23.00 (15.41–30.00)	24.20 (18.10–30.37)	4.20 (2.50–7.11)	0.601 (0.506–0.715)	<0.001
Neur (%)	64.64 (56.28–73.04)	63.47 (55.31–70.34)	93.50 (87.40–95.18)	1.348 (1.228–1.479)	<0.001
ALT (u/l)	37.5 (25–57)	38 (25–56.75)	37 (27–65)	1.003 (0.999–1.006)	0.126
AST (u/l)	31 (23–41)	30 (23–38)	54 (47.5–73.75)	1.002 (0.999–1.003)	0.052
ALB (g/l)	35.15 (31.40–38.80)	35.60 (32.40–39.43)	27.90 (25.40–29.60)	0.591 (0.501–0.697)	<0.001
Cr (μmol/l)	67.00 (54.20–79.75)	67.00 (54.20–79.00)	65.00 (46.70–86.00)	0.995 (0.979–1.011)	0.548
BUN (mmol/l)	3.60 (3.10–4.53)	3.50 (3.03–4.30)	9.79 (5.83–12.08)	1.810 (1.514–2.163)	<0.001
Na^+^ (mmol/l)	141 (139–143)	141 (139.7–143)	140.5 (138–144.5)	1.028 (0.935–1.130)	0.570
Ca^2+^ (mmol/l)	2.32 (2.22–2.39)	2.33 (2.24–2.39)	2.10 (1.99–2.12)	0.501 (0.168–1.494)	0.532
Cl^−^ (mmol/l)	103 (101–105)	103 (101–105)	101 (96–106.65)	1.004 (0.923–1.092)	0.921
K^+^ (mmol/l)	4.23 (3.90–4.60)	4.30 (4.00–4.52)	4.00 (3.50–4.90)	0.663 (0.328–1.341)	0.253
PT (s)	13.3 (12.9–14.2)	13.3 (12.9–14.0)	14.8 (14.0–16.6)	2.807 (1.896–4.154)	<0.001
FIB (g/l)	3.85 (3.13–4.85)	3.80 (3.20–4.54)	5.31 (3.05–5.99)	1.461 (1.074–1.989)	0.016
DD (μg/ml)	0.38 (0.30–0.88)	0.35 (0.30–0.74)	4.02 (2.34–9.84)	2.307 (1.729–3.078)	<0.001
CRP (mg/l)	6.15 (3.14–30.70)	4.13 (2.98–21.10)	126.52 (65.51–186.30)	1.032 (1.024–1.041)	<0.001
ESR (mm/h)	44 (25–70)	40 (22–70)	31 (12–79)	1.009 (0.972–1.046)	0.649

OR, odd ratio in single variable logistic regression analysis; Days, the duration from first symptoms to hospital admission; WBC, white blood cell; Lym, lymphocyte; Lymr, lymphocyte ratio; Neu, neutrophil; Neur, neutrophil ratio; ALB, albumin; ALT, alanine aminotransferase; AST, aspartate amino transferase; BUN, blood urea nitrogen; Cr, creatinine; Ca2 + , calcium; Cl-, chlorine; K + , potassium; Na + , sodium; DD, D-dimer; FIB, fibrinogen; PT, prothrombin time; CRP, c-reactive protein; ESR, erythrocyte sedimentation rate.

## Results

(1) Characteristics of patients

The study included 336 adult patients with severe or critically ill COVID-19 eventually. The median age was 65 years (IQR, 51–69), and 169 (50.3%) were men. The median duration from first symptoms to hospital admission was 2 days (IQR, 1–4). In a total of 336 patients, 182 (54.2%) had one or more underlying conditions. In all, 34 (10.1%) patients died during hospitalisation while 302 (89.9%) were discharged. Compared with survivors, non-survivor patients were significantly older with a median age of 74 years (IQR, 64–78) *vs.* 64 years (IQR, 51–68) (Table 1).

(1) Laboratory findings

Laboratory parameters of all patients were recorded on day of hospital admission, then divided into survivor or non-survivor groups according to their clinical outcome. The levels of WBC count, neutrophil (Neu), neutrophil ratio (Neur), blood urea nitrogen (BUN), D-dimer (DD) and c-reactive protein (CRP) were higher in non-survivor patients, together with the reduction of lymphocyte ratio (Lymr) (Table 1).

(1) Results of logistic regression analysis

In single variable analysis, age, WBC count, Lymr, Neur; serum albumin (ALB), BUN; prothrombin time (PT), fibrinogen (FIB), DD; and CRP were associated with death (Table 1). WBC, Lym, Lymr, are all related to the WBC count, and may affect each other or have contained relationships. To avoid bias in the prediction model due to correlation, it is better for the variables in a model to be completely independent of each other. Similarly, FIB, PT and DD all reflected the coagulation function, and we chose only one to represent it. Therefore, we excluded WBC, PT and FIB in the subsequent analysis. This was followed by multivariable regression analysis using the backward stepwise method (likelihood ratio), and variables entered in this step included DD, ALB, Age, Lymr, Neur, BUN, CRP. The results of the analysis are presented in Table 2. Eventually, we found that Lymr, BUN and DD at admission were closely related to death.

(1) Combination of predictors and development of predictive model

**Fig. 2.**
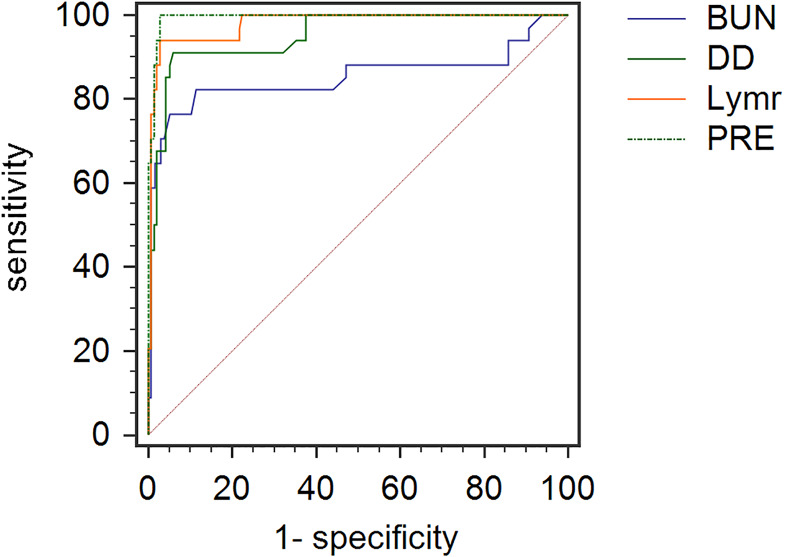
ROC curves of Lymr, BUN, D-dimer and PRE. ROC, receiver operating characteristic; Lymr, lymphocyte ratio; BUN, blood urea nitrogen; DD, D-dimer; PRE, a combined predictive factor with the three factors above.

Three laboratory indicators were combined to provide a predictive probability value for the outcome of death in COVID-19 patients, which was expressed in terms of PRE. The ROC curve was then used to evaluate the predictive efficiency of the combined predictor and individual factors for the outcome of death, which is shown in Figure 2. According to it, the area under the curve (AUC) and cut-off values of the three factors were calculated (Table 3). As demonstrated, the optimal thresholds of Lymr, BUN and D-dimer were 8.615%, 5.95 mmol/l and 1.56 μg/ml.

**Table 2.** Result of backwards stepwise logistics regression

Variables	*B*	s.d.	Wald	*P*	OR (95% CI)
DD	0.288	0.091	9.911	0.002	1.334 (1.115–1.596)
Lymr	−0.555	0.134	17.051	0.000	0.574 (0.441–0.747)
BUN	0.414	0.159	6.801	0.009	1.513 (1.108–2.065)
Constant	1.250	1.061	1.389	0.239	3.491

*B,* the coefficient value of the constant term or variable in the regression model; s.d., standard error; Wald, the *χ*^2^ value of regression coefficient or constant term in Wald test; OR, odds ratio; DD, D-dimer; Lymr, lymphocyte ratio; BUN, blood urea nitrogen.

**Table 3.** AUC of meaningful factors predicting death

	AUC (95% CI)	Cut-off value	Sensitivity	Specificity	PPV	NPV
Lymr (%)	0.980 (0.961–0.999)	8.615	0.941	0.972	0.800	0.993
BUN (mmol/l)	0.853 (0.755–0.951)	5.950	0.765	0.948	0.634	0.973
DD (μg/ml)	0.951 (0.916–0.987)	1.560	0.912	0.941	0.646	0.990
PRE	0.994 (0.979–0.999)	0.115	1.000	0.972	0.810	1.000

AUC, area under the curve; NPV, negative predictive value; PPV, positive predictive value; Lymr, lymphocyte ratio; BUN, blood urea nitrogen; DD, D-dimer; PRE, a combined predictive factor with the three factors above.

On the basis of the logistic regression model and the ROC curves, the scoring system for prediction of death was developed with three variables including Lymr, BUN and DD. When Lymr < 8.615%, 1 point is counted, otherwise 0 points are counted. Similarly, when BUN ≥ 5.95 mmol/l or D-dimer ≥ 1.56 μg/ml, 1 point is counted, otherwise 0 points are counted. The total score for each patient was calculated by summing points of each risk factor. We also drew the ROC curve of the combination for predicting risk of death in patients with severe or critically ill COVID-19 in Figure 2. The combined prediction model had the AUC of 0.994 (95% CI 0.979–0.999), with specificity/sensitivity of 97.24%/100.00% and positive predictive value (PPV)/negative predictive value (NPV) of 81.0%/100.0% (Table 3). The cut-off value of it was 0.115.

(1) Comparison of the above three indicators of patients who died at different time points

**Fig. 3.**
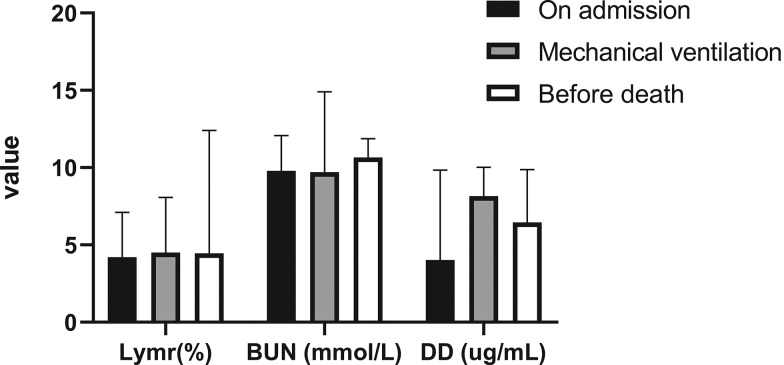
Meaningful indicators of patients who died at different time points. Lymr, lymphocyte ratio; BUN, blood urea nitrogen; DD, D-dimer.

To further investigate the changes in D-dimer, BUN and Lymr in the progression of disease in patients who died, we compared these three indicators at three different time points (the beginning of hospital admission, the beginning of mechanical ventilation and before death). Totally, 36 patients were ever treated with mechanical ventilation (invasively or non-invasively), including those 34 cases of death. That is, all patients who died underwent mechanical ventilation during treatment. As shown in Table 4 and Figure 3, the results showed no significant differences among the three time points for these indicators. For example, BUN was not significantly different in patients who died at the beginning of hospital admission, at the beginning of mechanical ventilation and before death, which means this indicator did not change significantly during the progression of disease in patients who died.

**Table 4.** Meaningful indicators of patients who died at different time points

	On admission	Mechanical ventilation	Before death	*P*-value
Lymr (%)	4.20 (2.50–7.11)	4.50 (2.38–8.08)	4.45 (1.95–12.4)	0.568
BUN (mmol/l)	9.79 (5.83–12.08)	9.71 (5.28–14.90)	10.65 (8.75–11.87)	0.361
DD (μg/ml)	4.02 (2.34–9.84)	8.16 (6.43–10.02)	6.45 (3.03–9.87)	0.121

Lymr, lymphocyte ratio; BUN, blood urea nitrogen; DD, D-dimer.

*Notes*: 95% confidence interval in brackets.

## Discussion

Since the outbreak of COVID-19, many studies have revealed its virological characteristics, transmission characteristics, clinical manifestations, but there were few studies on patients who died. It is now clear that SARS-CoV-2, a kind of coronavirus, belongs to the *β*-coronavirus family, and could cause severe coronavirus disease similar to severe acute respiratory syndrome (SARS) and Middle East respiratory syndrome (MERS) [17]. At the same time, based on previous research, the virus can spread rapidly from person to person. Its basic reproductive number (*R*_0_) is estimated to be 2.2, which means that each patient has transmitted the infection to 2.2 other people on an average [4]. The clinical manifestations of the disease are variable. In previous studies, most patients had mild symptoms such as fever, fatigue and dry cough [8], with lower overall mortality than SARS and MERS [11], but the mortality rate of severe patients was higher than those two diseases [9]. Therefore, in this research study, we analysed the laboratory examination indicators of patients who died and hoped to find out the risk factors that could predict the outcome of death. Through analysis, we screened three indicators of DD, lymphocyte ratio and BUN as predictors of the outcome of death.

DD, as a commonly used clinical and simple test, effectively reflects the activation of the coagulation system. In our study, DD higher than 1.56 μg/ml was closely related to fatal outcome of COVID-19. It has been proved that high levels of DD are significantly related to 28-day fatality in patients with infection or sepsis detected in the emergency department [18]. The cohort study from Jin Yin-tan Hospital and Wuhan Pulmonary Hospital (Wuhan, China) also found that DD greater than 1 μg/ml was the risk factor of death in COVID-19 patients. Meanwhile, DD is an activation marker of coagulation cascade, which is considered an early event in patients with infection and sepsis [19]. Therefore, elevation of DD may indicate severe infection or sepsis, which is often the cause of death in patients with COVID-19.

As mentioned in previous studies, lymphocytopenia occurred in most severe patients [8, 9]. This laboratory abnormality is similar to it previously observed in patients with SARS and MERS [20, 21]. Consistent with these research studies, our findings showed that lymphocyte ratio lower than 8.615% was highly associated with death of COVID-19 patients. Therefore, we speculated that the virus may mainly affect lymphocytes, leading to their apoptosis, thereby triggering immune dysfunction in patients, which may be the reason why some patients rapidly develop sepsis and multiple organ failure. Some studies have shown that a drastic reduction in the total number of lymphocytes indicates that the coronavirus could consume many immune cells and suppressed the cellular immune function [22], suggesting that injury of T lymphocytes may be an important factor leading to worsening of the patient's condition. Hence, application of immunomodulators may improve infection status in critically ill patients.

BUN often implies acute kidney injury and is also considered an important predictor of organ failure. Our analysis showed that elevated blood urea was closely related to the poor prognosis of patients with COVID-19, which was also mentioned briefly in other studies [8]. We suspected that the elevation of BUN may be related to acute kidney injury, which may be caused by the invasion of the virus itself, insufficient tissue oxygen supply and shock.

In other research studies, older age has been reported as a favourable risk factor of mortality [9, 13], but through multivariate analysis, our study excluded age as a predictor of death. The reason was that the main objective of previous research was the comparison between severe and non-severe patients, but our study mainly focused on the cases of death in severe patients, in which case the effect of age may be relatively small. We further collected laboratory indexes performed during the hospitalisation of these patients who died and before their death. The timing of mechanical ventilation in these deceased patients was also the time of their worsening condition, so we chose to retrospectively analyse changes in the corresponding indicators at these time points in order to explore the relationship between these indicators and progression of disease. By comparing the above three indicators at different time points, we found that the difference was not significant (Table 4, Fig. 3). This may mean that relevant indicators of these patients have not changed obviously from the early stages of admission to the clinical outcome, and existing treatment may not significantly slow the deterioration of the disease. It also implies that these deceased patients showed signs of developing a severe outcome at the beginning of hospital admission, indicating the rationality of our prediction using the initial admission data as well as suggesting that patients with this trend could be screened out early and need to be focused on treatment to reduce the COVID-19 morbidity rate.

To our knowledge, this is the largest number of studies involving severe COVID-19 patients, and it is also one of the few studies focusing on patients who died. Compared with single-factor predictions in other studies [13], our designed multifactor scoring system is significantly more accurate. The ROC curve also shows that this method has extremely high specificity and sensitivity. At the same time, the three indicators required by this prediction method are easy to obtain at the time of admission and can be implemented in other medical centres.

Our study also has some limitations. Firstly, there is a certain bias due to retrospective research. Some indicators that may be meaningful are not routinely tested in the clinic, such as cytokines, lactic acid, transferrin and so on. Secondly, the research sample size is still too small. It is still a single-centre study and could not represent the overall situation well. Thirdly, the scoring system is established and evaluated using the same group of patients, which makes the evaluation results may be less accurate, and a prospective research cohort needs to be established to further validate its accuracy.

## Conclusion

We found that three factors including decreased lymphocyte ratio, elevated BUN and raised D-dimer were related to death outcomes in critically ill patients with COVID-19. A combined multifactorial prediction model with high accuracy was developed to predict the clinical outcome of these patients.

## Data Availability

The data that support the findings of this study are available from Union Hospital, Tongji Medical College, Huazhong University of Science and Technology. Restrictions apply to the availability of these data, which were used under licence for this study.
